# Chromosome Arm Locations of Barley Sucrose Transporter Gene in Transgenic Winter Wheat Lines

**DOI:** 10.3389/fpls.2019.00548

**Published:** 2019-04-30

**Authors:** Shotaro Takenaka, Winfriede Weschke, Bettina Brückner, Minoru Murata, Takashi R. Endo

**Affiliations:** ^1^Department of Plant Life Science, Faculty of Agriculture, Ryukoku University, Otsu, Japan; ^2^Department of Molecular Genetics, Leibniz Institute of Plant Genetics and Crop Plant Research (IPK), Gatersleben, Germany; ^3^Department of Breeding Research, Leibniz Institute of Plant Genetics and Crop Plant Research (IPK), Gatersleben, Germany; ^4^Faculty of Science, Universiti Tunku Abdul Rahman, Kampar, Malaysia

**Keywords:** HOSUT lines, wheat, barley sucrose transporter, transgene, nullisomic-tetrasomics, chromosome banding, telocentric chromosome

## Abstract

Three transgenic HOSUT lines of winter wheat, HOSUT12, HOSUT20, and HOSUT24, each harbor a single copy of the cDNA for the barley sucrose transporter gene *HvSUT1* (SUT), which was fused to the barley endosperm-specific Hordein B1 promoter (HO; the HOSUT transgene). Previously, flow cytometry combined with PCR analysis demonstrated that the HOSUT transgene had been integrated into different wheat chromosomes: 7A, 5D, and 4A in HOSUT12, HOSUT20, and HOSUT24, respectively. In order to confirm the chromosomal location of the HOSUT transgene by a cytological approach using wheat aneuploid stocks, we crossed corresponding nullisomic-tetrasomic lines with the three HOSUT lines, namely nullisomic 7A-tetrasomic 7B with HOSUT12, nullisomic 5D-tetrasomic 5B with HOSUT20, and nullisomic 4A-tetrasomic 4B with HOSUT24. We examined the resulting chromosomal constitutions and the presence of the HOSUT transgene in the F_2_ progeny by means of chromosome banding and PCR. The chromosome banding patterns of the critical chromosomes in the original HOSUT lines showed no difference from those of the corresponding wild type chromosomes. The presence or absence of the critical chromosomes completely corresponded to the presence or absence of the HOSUT transgene in the F_2_ plants. Investigating telocentric chromosomes occurred in the F_2_ progeny, which were derived from the respective critical HOSUT chromosomes, we found that the HOSUT transgene was individually integrated on the long arms of chromosomes 4A, 7A, and 5D in the three HOSUT lines. Thus, in this study we verified the chromosomal locations of the transgene, which had previously been determined by flow cytometry, and moreover revealed the chromosome-arm locations of the HOSUT transgene in the HOSUT lines.

## Introduction

In transgenic wheat lines carrying the cDNA for the barley sucrose transporter gene *HvSUT1* (SUT) fused to the Hordein B1 promoter (HO; the HOSUT transgene), *HvSUT1* is overexpressed and the uptake of sucrose into grains is increased because the Hordein B1promoter is highly active in maturing cereal endosperm (Weichert et al., 2010). All three HOSUT lines significantly increased grain yield under semi-controlled conditions, together with higher protein yield and higher iron and zinc concentration compared with the wild type cultivar (Saalbach et al., 2014). Identification of transgene insertion sites in genomes has practical implications for crop breeding. In Arabidopsis and rice, direct methods such as thermal asymmetric interlaced PCR (TAIL-PCR) have been successfully used to determine genomic DNA sequences flanking T-DNA inserts containing some marker genes (Liu et al., 1995; Liu and Chen, 2007). In wheat, however, it is still difficult to know the positions of transgenes by TAIL-PCR because of its large and complex genome. Cápal et al. (2016) applied flow cytometry to identify the chromosomal location of the transgene in the three HOSUT lines. They sorted the wheat chromosomes into individual chromosomes by flow cytometry and analyzed the flow-sorted chromosomes by PCR and fluorescence *in situ* hybridization (FISH), and found that each of the HOSUT lines had single insertion sites of the transgene on separate chromosomes, 4A, 7A, and 5B. Cápal et al. (2016) also performed whole genome amplification of single chromosomes that were flow-sorted from each of the HOSUT lines and confirmed the chromosomal locations of the HOSUT transgene in the HOSUT lines.

We were convinced that the HOSUT transgene chromosomal locations should be reconfirmed by independent approaches because the substantial yield increase in the HOSUT lines might be of considerable future interest for wheat breeding, and because the flow cytometry approach of Cápal et al. (2016) was used to localize a transgene to chromosomes for only the first time in wheat. At first, we tried single-copy FISH with the *HvSUT1* probe without success. Since the main purpose of this study was to confirm the authenticity of the chromosomal locations of the integrated transgene that had been provisionally established by flow cytometry, we decided to conduct a conventional aneuploid analysis using aneuploid lines of wheat, because this analysis is the most reliable approach to identifying chromosomes carrying genes of interest in wheat. In addition, telocentric chromosomes harboring the HOSUT transgene were expected to arise in the progeny of hybrids between the wheat aneuploid and HOSUT lines. Such telocentric chromosomes, which are smaller than any intact wheat chromosomes and can easily be sorted by flow cytometry, would be useful in future research on the chromosomal and DNA organization around the integration sites of the transgene. In this study we employed the nullisomic-tetrasomic lines of common wheat for aneuploid analyses, reconfirmed the chromosomal locations of the HOSUT transgene in the three independent HOSUT lines, and identified the chromosome arms carrying the HOSUT transgene integrations.

## Materials and Methods

### Plant Material and Cytology

**FIGURE 1:**
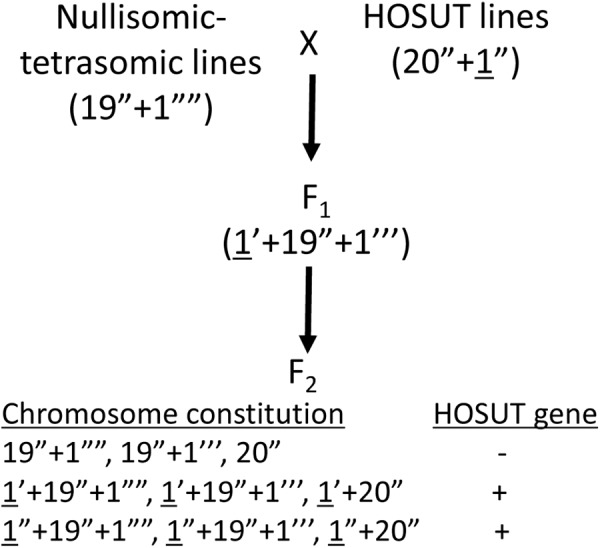
Crossing scheme to verify the critical chromosomes carrying the HOSUT transgene (underlined). F_2_ plants are expected to have various chromosome constitutions regarding the critical chromosomes. The transgene is expected to be present in the F_2_ plants carrying the critical chromosome (HOSUT gene +).

We used three transgenic lines of winter wheat (*Triticum aestivum* L., *2n* = 42, genome constitution AABBDD) cv. Certo: HOSUT12, HOSUT20, and HOSUT24, which had been used in the flow cytometry study by Cápal et al. (2016). Cápal et al. (2016) reported that the HOSUT transgene is located on chromosome 7A in HOSUT12, on chromosome 5D in HOSUT20, and on chromosome 4A in HOSUT24. Therefore, we cross-fertilized three nullisomic-tetrasomic lines of common wheat cv. Chinese Spring with the three HOSUT lines, namely nullisomic 7A-tetrasomic 7B (N7AT7D) with HOSUT12, nullisomic 5D-tetrasomic 5B (N5DT5B) with HOSUT20, and nullisomic 4A-tetrasomic 4B (N4AT4B) with HOSUT24. In nullisomic-tetrasomic lines, one pair of homologous chromosomes is replaced by an extra pair of homoeologous chromosomes (Sears, 1954). These F_1_ hybrids were self-fertilized to obtain F_2_ progeny. Figure 1 illustrates the process of cross- and self-fertilization, and the expected chromosome configurations in the progeny. Root tips and leaves were taken from all individuals of the F_2_ progeny for cytological and PCR analyses, respectively. We conducted the karyotyping of the F_1_ and F_2_ progeny, as well as cultivar Certo, by C-banding, following the protocols of Endo (2011). Individual chromosomes were identified based on the previously published banding karyotypes of common wheat (Endo and Gill, 1984; Gill et al., 1991).

### PCR

We conducted PCR analysis to demonstrate the presence or absence of the HOSUT transgene and critical chromosomes, namely 4A, 5D, or 7A, in the F_2_ progeny. One primer set for the HOSUT gene and two primer sets for each of the chromosomes were chosen from the list of PCR primers reported by Cápal et al., 2016; Table 1. DNA was extracted from young leaves using a DNeasy Plant Mini Kit (Qiagen Tokyo, Japan). The PCR mixture (15 μL) contained 30 ng of genomic DNA, 1 × Gflex PCR Buffer (TaKaRa, Japan), 0.5 μM primers and 0.375 U of Tks Gflex DNA Polymerase (TaKaRa, Japan). PCR conditions were 94°C for 1 min followed by 30 cycles of 98°C for 10 s, 60°C for 15 s, and 68°C for 30 s. PCR products were separated on 3% agarose (w/v) gels in TAE buffer.

**Table 1:** PCR primer sets for the HOSUT gene and wheat chromosomes 4A, 5D, and 7A.

Primer set	Target	Forward (5′ → 3′)	Reverse (5′ → 3′)	Amplicon size (bp)	Annealing temperature (°C)
HvSUT_RT	HvSUT1	CGGGCGGTCGCAGCTCGCGTCTATT	CATACAGTGACTCTGACCGGCACACA	169	62
Owm121	4A	ATTGCCGTCGCGAACTAGA	CGGGACGAGCTTGACGAT	351	60
Owm167	4A	TTTTCTTGGTCAGTATAACCTGTTTTT	TGAGCAGAGAAAAATTTCCAAG	285	60
Owm180	5D	CGGACGAGCAGCAGTACC	GCAGATCGGCATAAATTGAATGT	292	58
Owm184	5D	AGCATGCTCCCAAAGACTATTAC	GTTATGATGGTGGTAGCAATTTGA	400	58
Owm186	7A	CTCTCTGTGGCCAATAGTGC	TCTATACCTCAACCCTACATCCA	112	58
Owm190	7A	CGCATGGACATTGTTCTAGTCA	GCACTTAGGCACGCTTGAG	517	58

*The primer information was according to Cápal et al. (2016). Chromosome-specific Owm markers were designed by Primer3 based on the chromosome sequences from the International Wheat Genome Sequencing Consortium (IWGSC), while preventing the primers from amplifying the sequence from the homoeologous chromosomes.*

## Results and Discussion

**FIGURE 2:**
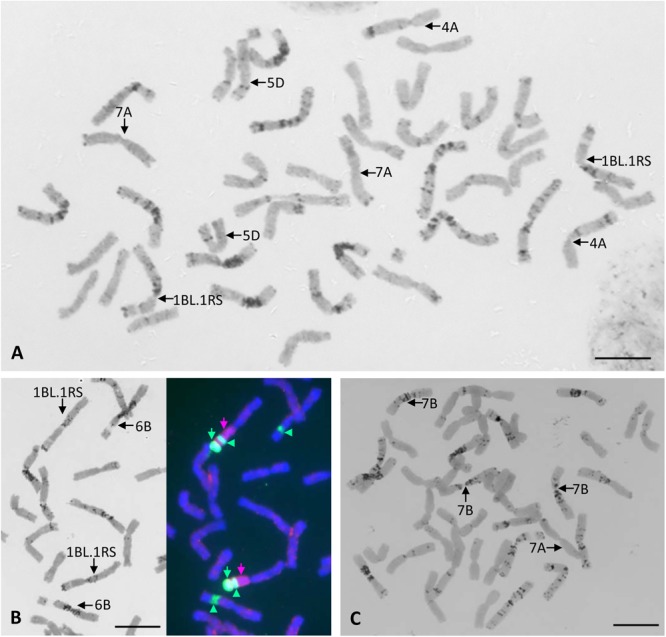
Chromosome constitutions of a common wheat cultivar Certo and an F_1_ hybrid between N7AT7B and HOSUT12. Chromosomes 4A, 7A, and 5D of Certo are similar to those of Chinese Spring wheat in terms of the C-banding pattern, and there is no wheat chromosome 1B in Certo **(A)**. Sequential C-banding-FISH/GISH shows that Certo is disomic for 1BL.1RS translocation substituting for chromosome 1B. Probes for FISH/GISH are rye total genomic DNA (indicated with pink arrows), pSc 200 sequences (indicated with green arrows), and 18S.26S rDNA (indicated with green arrowheads at the secondary constrictions). **(B)**. In the F_1_ hybrid there was only one chromosome 7A from HOSUT12 and three doses of chromosome 7B. **(C)**. Bar = 10 μm.

**FIGURE 3:**
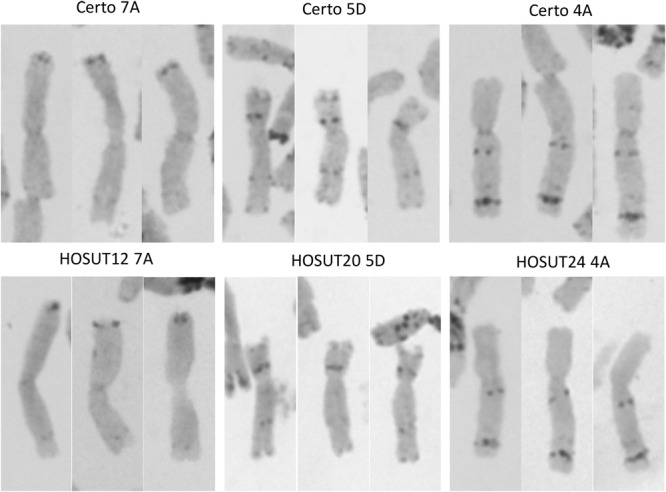
C-banding images (three for each) of chromosomes 7A, 4A, and 5D derived from Certo (upper row) and the HOSUT lines (lower row). Note that there was no obvious structural difference between the normal and transgene-carrying homologous chromosomes.

All chromosomes of Certo were identified based on their C-banding patterns (Figure 2A). The C-banding patterns of some of the Certo chromosomes were obviously different from those of Chinese Spring wheat, generally accepted as the standard cultivar for cytogenetic research with wheat, (Gill et al., 1991). Cultivar Certo had a wheat-rye translocation substituting for chromosome 1B, probably a translocation between the long arm of chromosome 1B and the short arm of rye chromosome 1R (1BL.1RS) because the C-banding pattern of its long arm was similar to that of chromosome 1B and because FISH/GISH detected rye-specific pSc200 subtelomeric and genomic chromatin signals in its short arm (Figure 2B). Although the banding patterns of Certo chromosomes 2B, 3B, and 7B were different from those of Chinese Spring chromosomes 2B, 3B, and 7B, they were identified by consulting the chromosome banding patterns of other wheat cultivars (Endo and Gill, 1984). The C-banding analysis confirmed that the F_1_ hybrids between the HOSUT lines and nullisomic tetrasomic lines were monosomic for the critical chromosome and trisomic for the respective homoeologous chromosomes (Figure 2C). Chromosome constitutions were successfully identified in all F_2_ seedlings (Table 2). As far as C-banding patterns are concerned, there was no structural difference between the Certo and HOSUT homologous chromosomes 7A, 5D, and 4A (Figure 3).

**Table 2:** Number of F_2_ plants with (+) or without (-) the critical chromosome (C), the PCR markers for the critical chromosome (M1, M2) and *HvSUT1* (H).

F_2_ of the following crosses	C+/M1+/ M2+/H+	C-/M1+/ M2-/H+	C-/M1-/ M2-/H-
N7AT7B × HOSUT12	32	0	10^a^
N5DT5B × HOSUT20	17	1^b^	11
N4AT4B × HOSUT24	25	3^c^	2

*^*a*^One of them was monotelosomic for 7AS. ^*b*^This plant was monotelosomic for 5DL. ^*c*^Two of them were monotelosomic for 4AL and the remaining one was hemizygous for a translocation involving 4AL.*

### F_2_ of N7AT7D × HOSUT12

**FIGURE 4:**
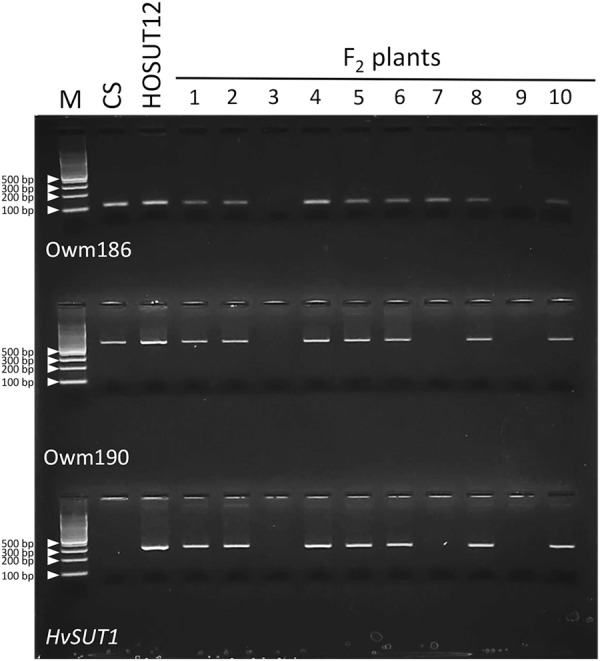
PCR analysis of part of the F_2_ progeny from a cross between N7AT7B and HOSUT12. The *HvSUT1* marker was not amplified in F_2_ plants 3, 7, and 9. Neither of the 7A-specific markers (Owm186 and Owm 190) were amplified in F_2_ plants 3 and 9, while Owm186 was amplified in F_2_ plant 7, which was identified by C-banding to be monotelosomic 7AS (Supplementary Figure S2). This result suggested that the *HvSUT1* marker was located on the long arm of chromosome 7A.

Among 42 F_2_ plants from the cross between N7AT7D and HOSUT12, 32 had intact chromosome 7A in the disomic condition (five plants) or in the monosomic condition (27 plants). The remaining 10 plants had no chromosome 7A (Table 2 and Supplementary Figure S1). Subsequent PCR analysis demonstrated that all of the 32 plants with chromosome 7A had both 7A-specific markers (Owm186 and 190) and the *HvSUT1* marker, and that the remaining 10 plants, with no chromosome 7A, had none of the three markers (Table 2 and Figure 4). This perfect concurrence of chromosome 7A and the *HvSUT1* marker clearly showed that the HOSUT transgene was located on chromosome 7A. One of the 10 F_2_ plants without the *HvSUT1* marker was monotelosomic for the short arm of chromosome 7A (7AS) and trisomic for chromosome 7B (Supplementary Figure S2). In this plant, one of the two 7A-specific markers (Owm190) was not amplified by PCR (Figure 4). This result suggested that Owm186 and Owm190 were located on the short and long arms of chromosome 7A, respectively, and that the 7A long arm carried the HOSUT transgene.

Assuming the HOSUT transgene was located on one of the other chromosomes, and that the transmission rate of the HOSUT transgene to the F_2_ progeny was 75%, as expected from the Mendelian segregation ratio in F_2_ progeny for a monohybrid cross, the probability that the transgene was transmitted to none of the 32 plants would be 0.0011(chi-square test). Therefore, it could statistically be deduced that the transgene was located on no other chromosome than chromosome 7A.

### F_2_ of N5DT5B × HOSUT20

**FIGURE 5:**
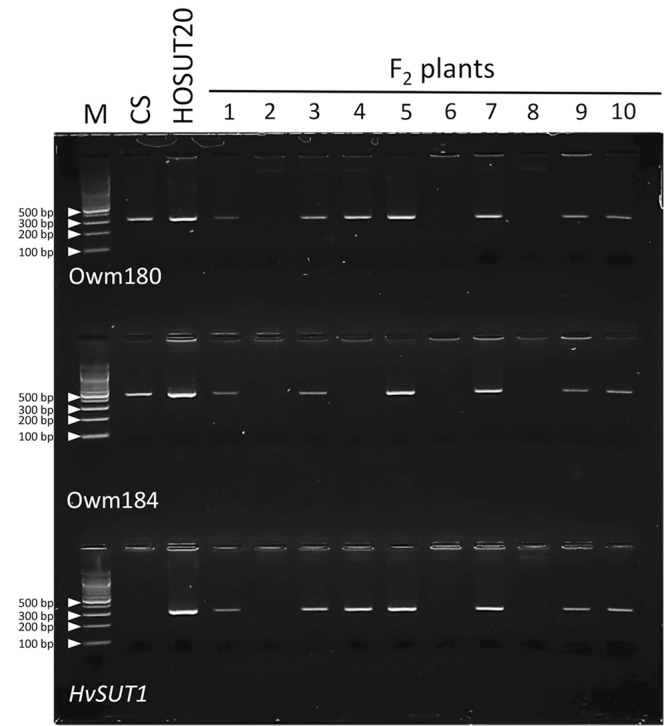
PCR analysis of part of the F_2_ progeny from a cross between N5DT5B and HOSUT20. The *HvSUT1* and two 5D-specific markers (Owm180 and Owm184) were not amplified in F_2_ plants 2, 6, and 8. In F_2_ plant 4, which was identified by C-banding to be monotelosomic 5DL (Supplementary Figure S4), the *HvSUT1* and Owm180 markers were amplified, but Owm184 was not. This result suggested that the *HvSUT1* marker was located on the long arm of chromosome 5D.

Among 29 F_2_ plants from the cross between N5DT5B × HOSUT20, 17 were either monosomic (11 plants) or disomic (6 plants) for chromosome 5D, 11 were nullisomic for chromosome 5D, and one was monotelosomic for the long arm of chromosome 5D (5DL) (Table 2 and Supplementary Figures S3, S4). Subsequent PCR analysis showed that all the plants monosomic or disomic for chromosome 5D had the *HvSUT1* marker as well as both 5D-specific markers (Owm180 and Owm184). On the other hand, the 11 nullisomic-5D plants had no *HvSUT1* marker and neither of the 5D-specific markers (Figure 5 and Table 2). The monotelosomic-5DL plant had the *HvSUT1* and Owm180 markers but not the Owm184 marker. This perfect association between the presence of chromosome 5D or 5DL and the *HvSUT1* marker suggested that the HOSUT transgene was located on the 5DL chromosome arm. At the same time, Owm180 and Owm184 were confirmed to be located on the long and short arms of chromosome 5D, respectively.

With similar reasoning, as mentioned above for the “F_2_ of N7AT7D × HOSUT12” progeny, the probability that the HOSUT transgene was transmitted to none of the 18 plants carrying chromosome 5D or 5DL is 0.0143 (chi-square test). Therefore, the null hypothesis that the transgene is located on a chromosome other than chromosome 5D can be rejected.

### F_2_ of N4AT4B × HOSUT24

**FIGURE 6:**
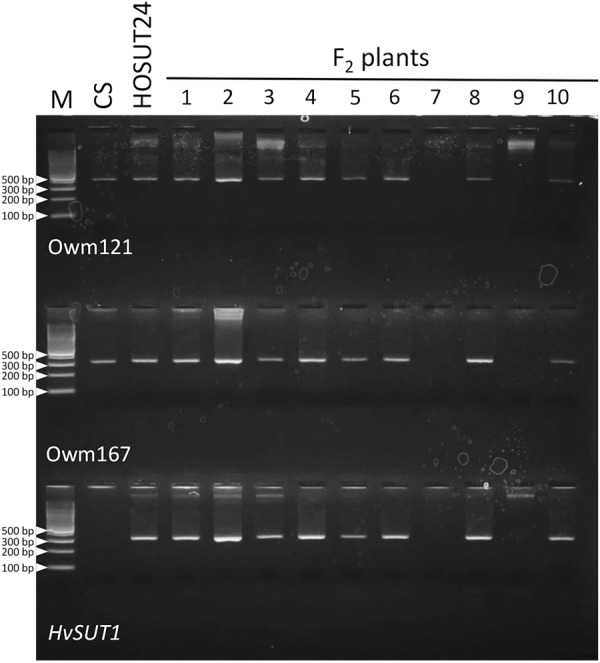
PCR analysis of part of the F_2_ progeny from a cross between N4AT4B and HOSUT24. The *HvSUT1* and two 4A-specific markers (Owm121 and Owm167) were not amplified in F_2_ plants 7 and 9. All three markers were amplified in F_2_ plant 3, which was identified by C-banding to be monotelosomic 4AL (Supplementary Figure S6). This result suggested that the *HvSUT1* marker was located on the long arm of chromosome 4A.

Among 30 F_2_ plants from the cross between N4AT4B × HOSUT24, 25 were either monosomic (23 plants) or disomic (two plants) for chromosome 4A, and two were nullisomic for chromosome 4A (Table 2 and Supplementary Figure S5). Two of the remaining three plants were monotelosomic for the long arm of chromosome 4A (4AL) (Supplementary Figure S6), and one had a translocation between the 4AL arm and the short arm of chromosome 6B (6BS) (Supplementary Figure S7). Subsequent PCR analysis showed that all of the 25 plants carrying the 4A chromosome and three plants carrying the 4AL arm had the *HvSUT1* and both 4A-specific markers. On the other hand, the two plants without chromosome 4A had none of the three markers (Figure 6 and Table 2). This perfect concurrence of chromosome 4A (or 4AL) and the HOSUT transgene suggested that the HOSUT transgene was located on the 4AL arm.

The low occurrence of nullisomics for chromosome 4A (6.7%), compared with the occurrence of nullisomics for chromosomes 7A (23.8%) and for chromosome 5D (37.9%), was probably due to inadequate compensation for the loss of chromosome 4A by two doses of chromosome 4B in pollen. It is known that there are rearrangements among chromosomes 4A, 5A, and 7B of modern-day hexaploid bread wheat, and that chromosome 4A carries translocated segments from chromosomes 5A and 7B (Devos et al., 1995). This fact suggests that the loss of chromosome 4A was inadequately compensated by the extra dose of chromosome 4B in this experiment.

Again, making a similar calculation to the one that we performed for the “F_2_ of N7AT7D × HOSUT12” progeny, the probability that the HOSUT transgene was transmitted to none of the 28 plants carrying chromosome 4A or 4AL is 0.0026 (chi-square test). Therefore, the null hypothesis that the transgene is located on a chromosome other than chromosome 4A can be rejected.

Taken together, the present study confirmed the chromosomal locations of the HOSUT transgene in the HOSUT lines as suggested by Cápal et al. (2016). FISH is the fastest way to identify transgene insertion sites in chromosomes, when it works. Although there have already been several studies reporting successful FISH of single-copy genes or cDNAs or transgenes in wheat (e.g., Anand et al., 2003; Danilova et al., 2012, 2014), we failed to assign the HOSUT gene to chromosomes by FISH. Therefore, the conventional aneuploid analysis is still the surest way of assigning specific genes or DNA sequences to specific chromosomes, although it is laborious.

In this study, telocentric chromosomes 5DL and 4AL carrying the transgene appeared in the F_2_ progeny of crosses between the nullisomic-tetrasomic lines and HOSUT lines. The size and morphology of telocentric chromosomes are good landmarks, which can serve to identify them under a microscope and isolate them from the chromosome complement by flow cytometry. After being established in telosomic lines, the 5DL and 4AL telocentric chromosomes can be flow-sorted onto microscope slides, which would make excellent, debris-free chromosome preparations for FISH analysis. In addition, flow-sorted chromosomes can be extremely stretched, sometimes more than seven times longer, than chromosomes prepared by the squash method (Endo et al., 2014). In producing the draft sequence of a hexaploid wheat genome, flow-sorted telocentric chromosomes were used for DNA extraction in order to reduce the complexity of the polyploid genome (International Wheat Genome Sequencing Consortium [IWGSC], 2014). Likewise, the 5DL and 4AL telocentric chromosomes harboring the transgene in the respective HOSUT lines can be flow-sorted for sequencing to analyze the DNA structure around the transgene insertion sites by various methods using next generation sequencing, such as targeted locus amplification (Cain-Hom et al., 2017). This strategy would be more efficient than performing whole genome sequencing or targeted locus amplification with the whole genome of the HOSUT lines.

## Author Contributions

TE conceived the plan of this study and wrote the manuscript. WW and BB cross-fertilized the HOSUT lines with the nullisomic-tetrasomic lines. MM grew and self-fertilized the F_1_ lines. TE and ST performed the cytological observation and PCR analysis, respectively. WW and MM revised the manuscript.

## Conflict of Interest Statement

The authors declare that the research was conducted in the absence of any commercial or financial relationships that could be construed as a potential conflict of interest. The reviewer AH declared a shared affiliation, though no other collaboration, with several of the authors WW and BB to the handling Editor.
