# Supplementary material for: Chromosome Arm Locations of Barley Sucrose Transporter Gene in Transgenic Winter Wheat Lines
Source: Front Plant Sci. 2019 Apr 30;10:548. doi: 10.3389/fpls.2019.00548 (PMC6502970; doi:10.3389/fpls.2019.00548)
Supplement: Supplementary file 1 [file Data_Sheet_1.PDF]

Supplementary Material

Chromosome Arm Locations of Barley Sucrose Transporter Gene in Transgenic Winter Wheat Lines

Shotaro Takanaka, Winfriede Weschke, Bettina Brückner, Minoru Murata, Takashi R. Endo*

*** Correspondence: Takashi R. Endo**: endo.takashi.2e@agr.ryukoku.ac.jp

Supplementary Figures

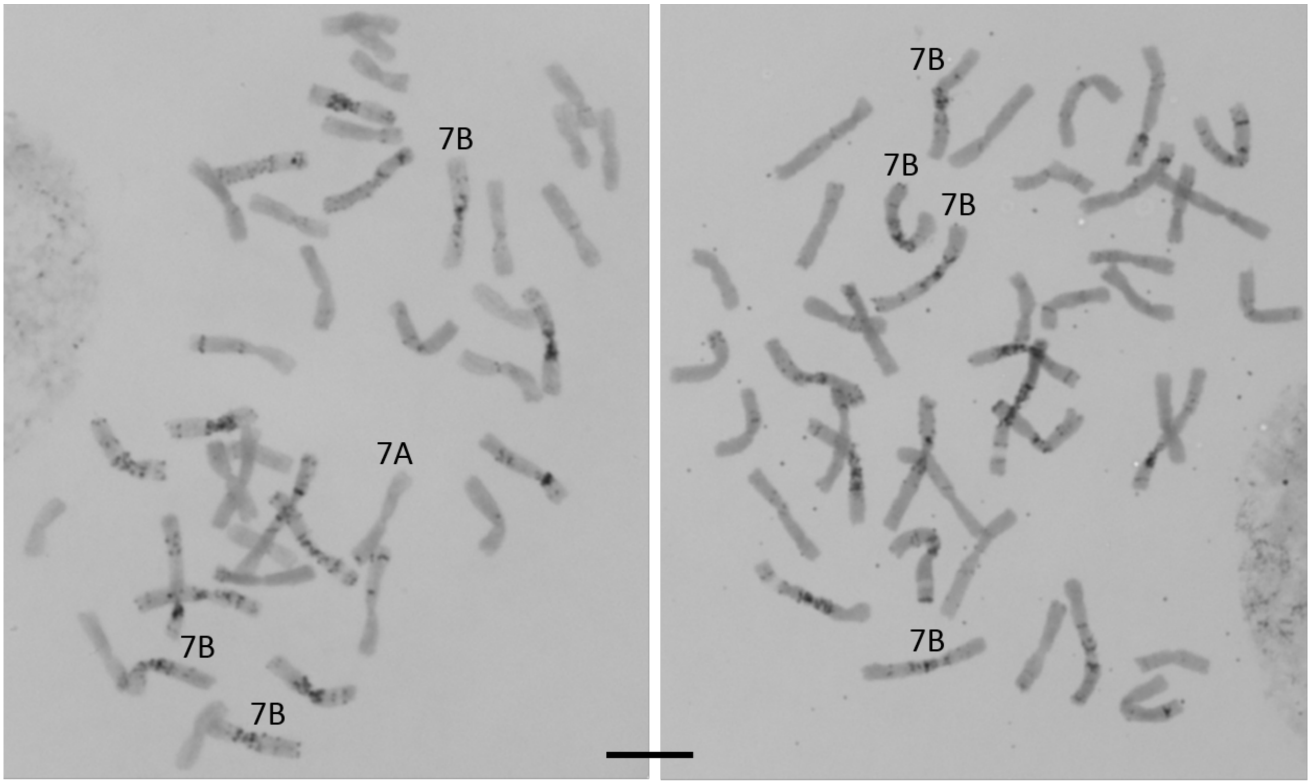

F_2: Monosomic 7A-trisomic 7B F_2: Nullisomic 7A-tetrasomic 7B

Supplementary Figure 1. C-banding images of two F_2 plants from the F_1 hybrid between N7AT7B and HOSUT12. One plant (left) was monosomic for 7A and trisomic for 7B, and the other plant (right) was nullisomic for 7A and tetrasomic for 7B. Bar = 10 µm.

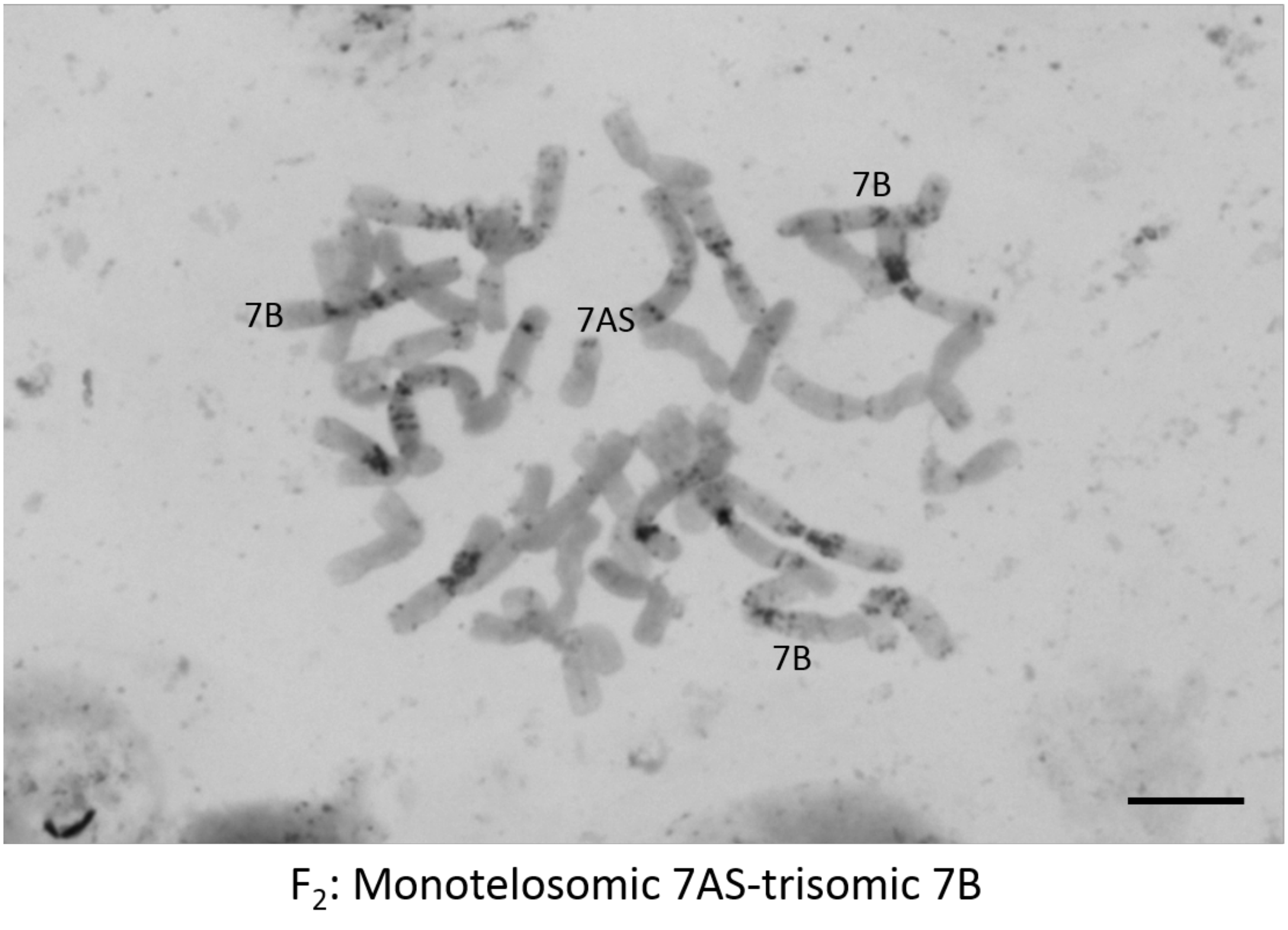

Supplementary Figure 2. C-banding image of an F_2 plant from the F_1 hybrid between N7AT7B and HOSUT12. This plant was monotelosomic for the short arm of chromosome 7A and trisomic for 7B. Bar = 10 µm.

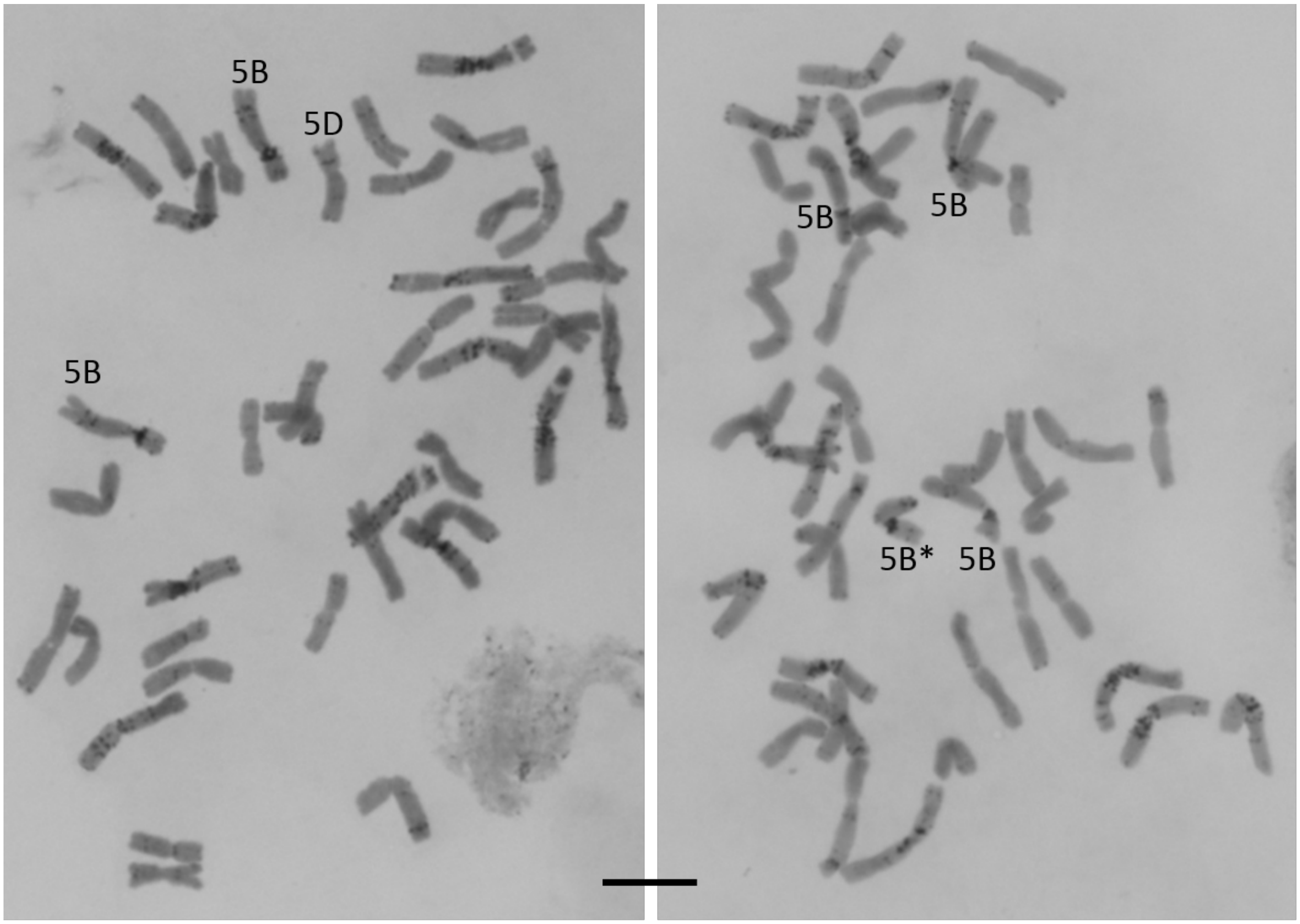

F_2 : Monosomic 5D-disomic 5B F_2 : Nullisomic 5D-tetrasomic 5B

Supplementary Figure 3. C-banding images of two F_2 plants from the F_1 hybrid between N5DT5B and HOSUT20. One plant (left) was monosomic for 5D and disomic for 5B, and the other plant (right) was nullisomic for 5D and partial trisomic for 5B. Note one of the 5B chromosomes had a deletion in the long arm (indicated with an asterisk). Bar = 10 µm.

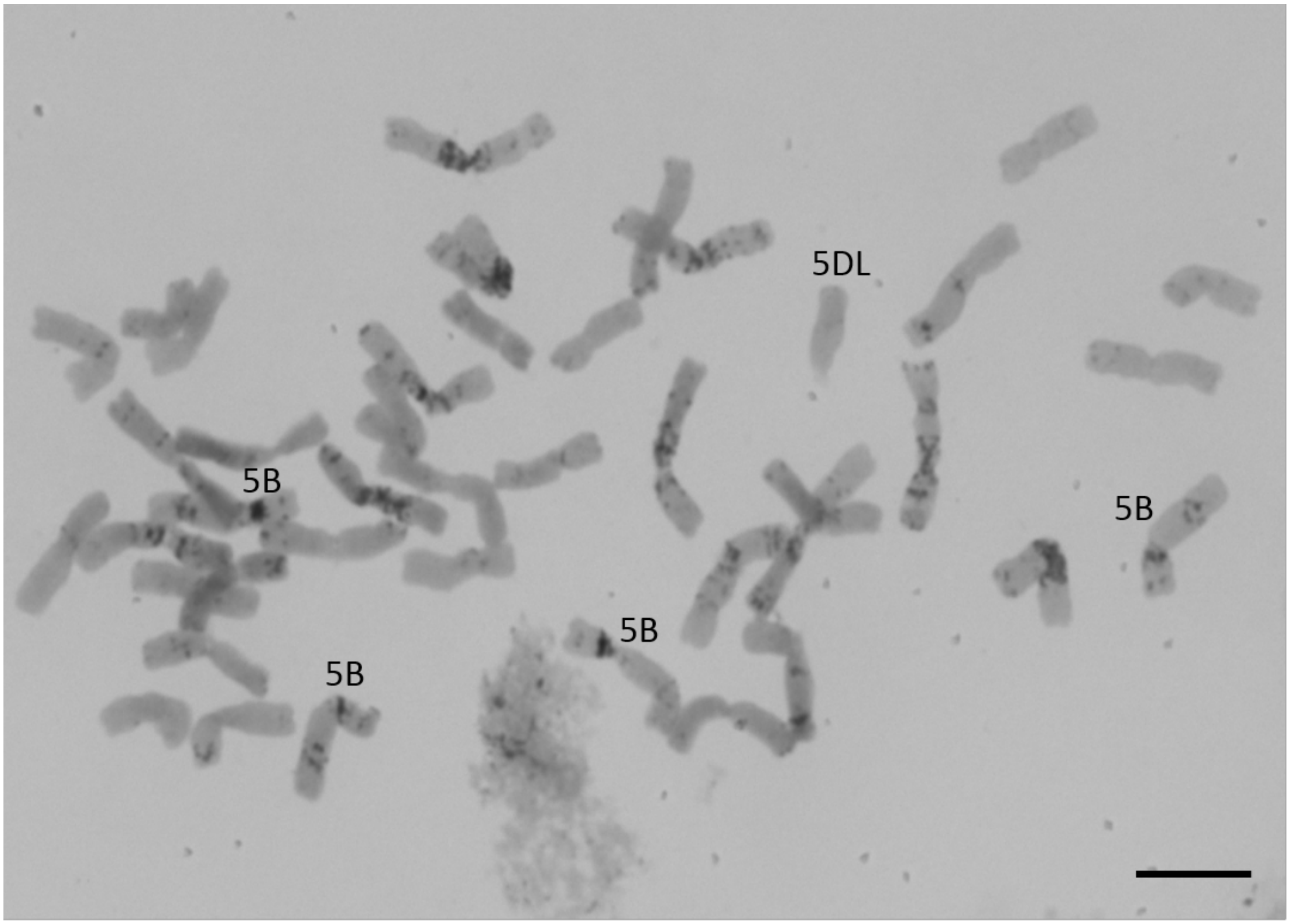

F$_2$: Monotelosomic 5DL-tetrasomic 5B

Supplementary Figure 4. C-banding image of an F_2 plant from the F_1 hybrid between N5DT5B and HOSUT20. This plant was monotelosomic for the long arm of chromosome 5D and tetrasomic for 5B. Bar = 10 µm.

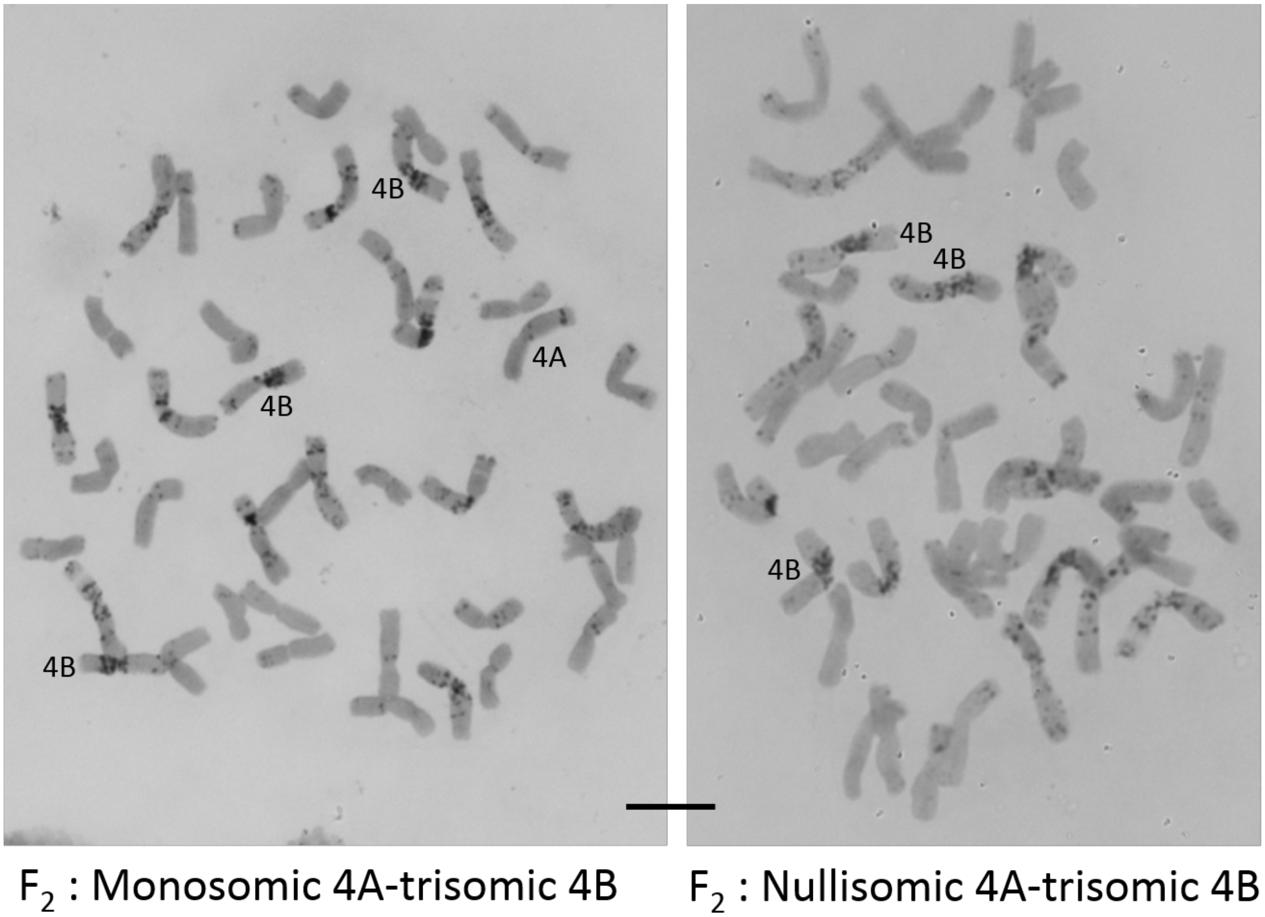

Supplementary Figure 5. C-banding images of two F_2 plants from the F_1 hybrid between N4AT4B and HOSUT24. One plant (left) was monosomic for 4A and disomic for 4B, and the other plant (right) was nullisomic for 4A and trisomic for 4B. Bar = 10 μm.

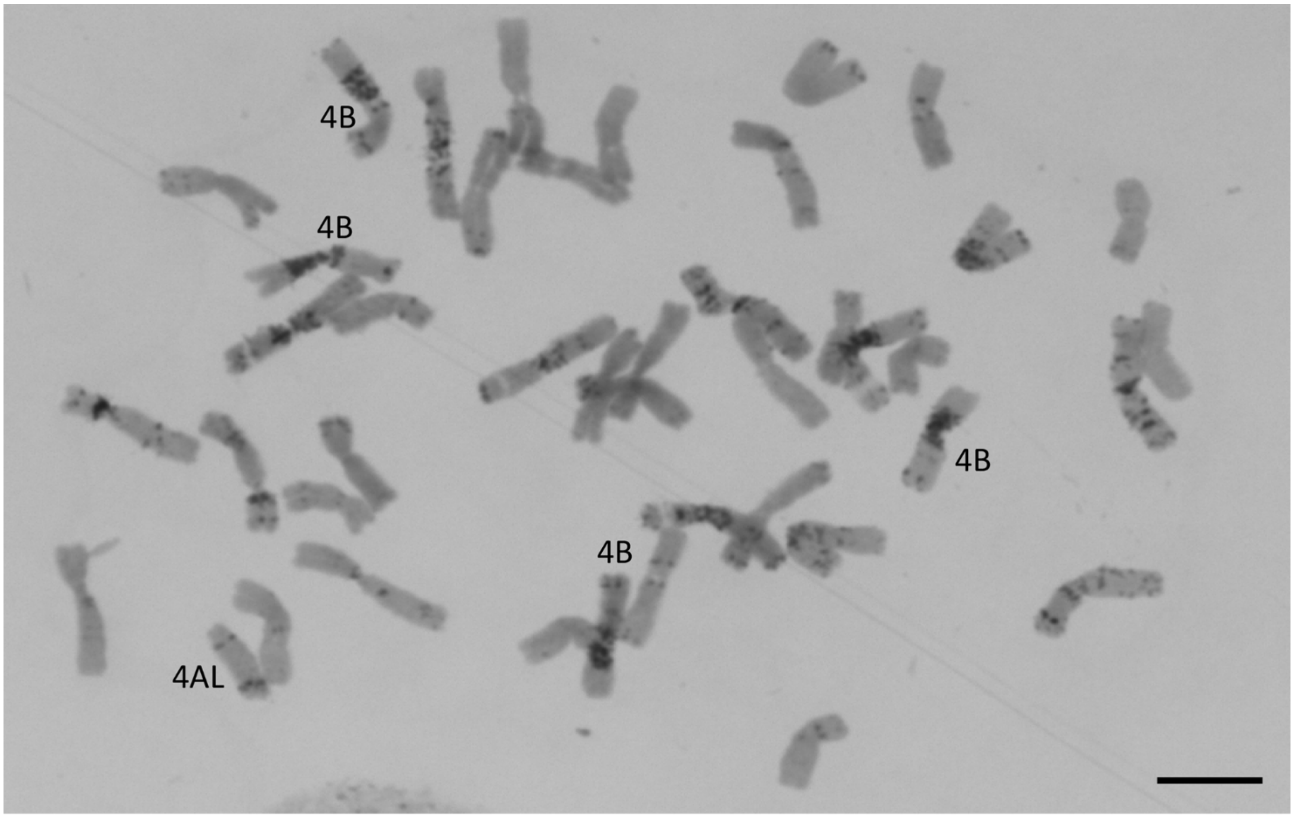

F_2 : Monotelosomic 4AL-tetrasomic 4B

Supplementary Figure 6. C-banding image of an F2 plant from the F1 hybrid between N4AT4B and HOSUT24. This plant was monotelosomic for the long arm of chromosome 4A and tetrasomic for 4B. Bar = 10 μm.

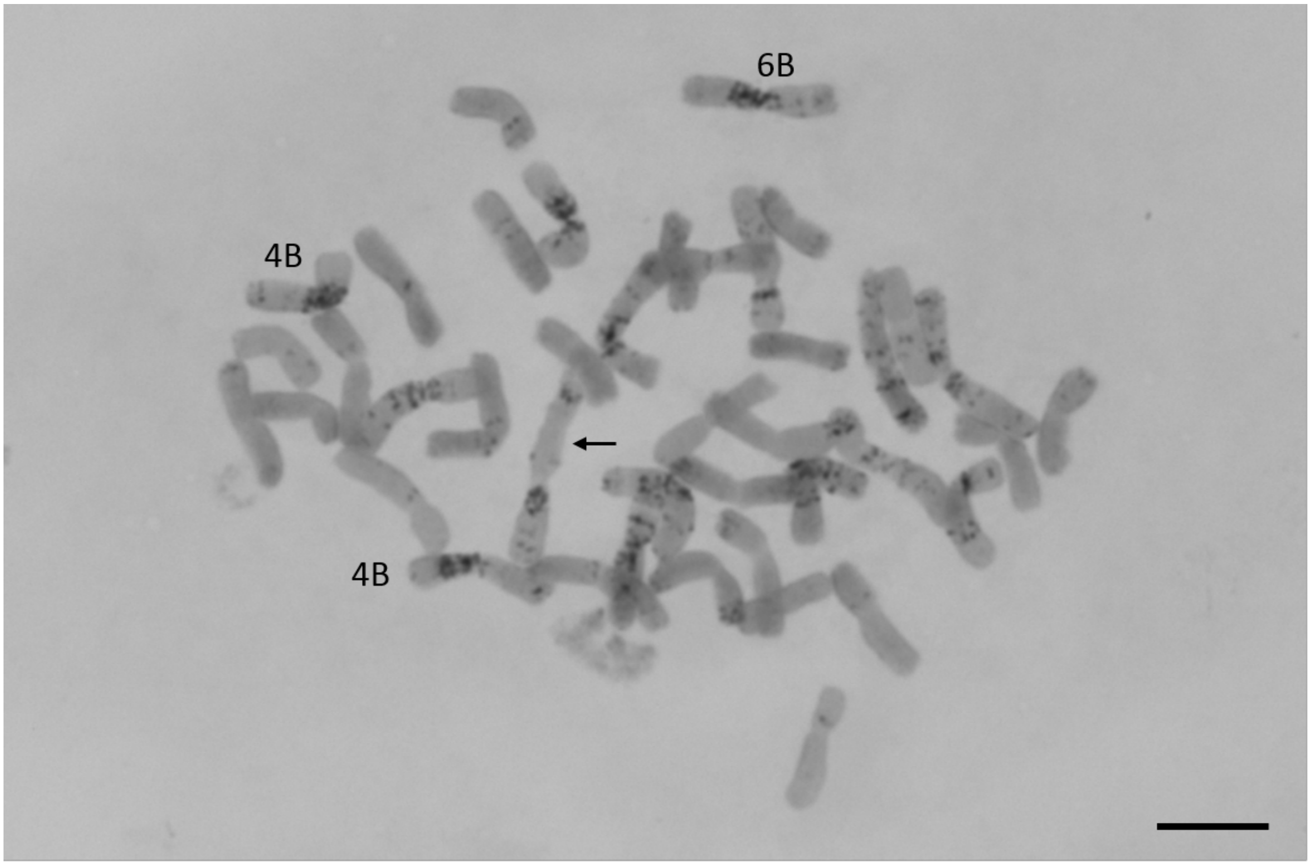

F$_2$: Translocation (6BS/4AL)

Supplementary Figure 7. C-banding image of an F_2 plant from the F_1 hybrid between N4AT4B and HOSUT24. This plant had no intact chromosome 4A, but instead it had a translocation between the short arm of chromosome 6B and the long arm of chromosome 4A (pointed with an arrow). Bar = 10 μm.
